# Mechanism of carrier controllability with metal capping layer on amorphous oxide SiZnSnO semiconductor

**DOI:** 10.1038/s41598-018-37530-6

**Published:** 2019-01-29

**Authors:** Byeong Hyeon Lee, Ahrum Sohn, Sangsig Kim, Sang Yeol Lee

**Affiliations:** 1 Department of Microdevice Engineering, Korea University, Seoul, 136-701 Korea; 2 Department of Semiconductor Engineering, CheongjuUniversity, Cheongju, 28503 Korea; 3 Research Institute of Advanced Semiconductor Convergence Technology, Cheongju, 28503 Korea; 4 School of Advanced Materials Science and Engineering, Sungkyunkwan University, Suwon, 16419 Korea

## Abstract

**The change of electrical performance of amorphous SiZnSnO thin film transistors (a-SZTO TFTs) has been investigated depending on various metal capping layers on the channel layer by causing different contact property. It was confirmed that the change of electrical characteristics was sensitively dependent on the change of the capping layer materials on the same channel layer between the source/drain electrodes. This sensitive change in the electrical characteristics is mainly due to different work function of metal capping layer on the channel layer. The work function of each capping layer material has been analyzed and derived by using Kelvin probe force microscopy and compared with the energy bandgap of the SZTO layer. When the work function of the capping layer is larger than that of the channel layer, electrons are depleted from the channel layer to the capping layer. On the contrary, in the case of using a material having a work function smaller than that of the channel layer, the electrical characteristics were improved because electrons were injected into the channel layer. Based on depletion and injection mechanism caused by different contact barrier between metal capping layer and channel layer, NOT, NAND, and NOR logic circuits have been implemented simply by changing metal capping layer on the channel layer**.

## Introduction

Amorphous oxide semiconductors (AOSs) have been applied to the next-generation electric and electronic industrial fields in recent decades. The AOS can be used as an active channel layer of thin film transistors (TFTs), which can be applied directly to organic light-emitting diodes and/or active-matrix liquid crystal display, which is a recent issue^1–4^. This is because AOS is composed of metal-oxide (M-O) network and the conduction band is composed of spherical ns orbitals, which shows high carrier mobility despite being amorphous. In addition, the AOS has a wide bandgap of 3 eV or more, which is transparent, and has a merit that can be applied widely from an insulator to a conductive materials^5,6^. AOS is known to control the density of state (DOS) in the energy bandgap by controlling the oxygen vacancy (*V*_*o*_) or the hydrogen content in the thin film^7,8^. To control this, an oxygen vacancy suppressor substance is added or an additional treatment process is used. Lee *et al*. recently reported the change of energy bandgap when Si was added on the ZTO (SZTO) system by DOS and X-ray photoelectron spectroscopy (XPS)^9,10^. Based on the reports, SZTO showed a systematic reduction of DOS and an increase in energy bandgap as Si content increased. This steady AOS study has contributed greatly to improving the quality of the thin film. When *V*_*o*_ is suppressed, the stability under various stresses, such as negative bias temperature illumination stress is improved but the conduction characteristics are reduced, which is complementary to each other^11,12^. Therefore, studies for improving electrical characteristics while securing stability have been actively conducted. Recently, many groups including Kim *et al*., Zan *et al*., and Choi *et al*. proposed a capping structure^13–15^ to secure the electrical characteristics and to improve the stability by depositing a conductive material between source/drain electrode. The capping layer structure improves the electrical properties and the stability of TFTs which could be explained by several mechanisms. According to the report of Kim *et al*., the electrical characteristics were improved by the movement of electrons during the contact between the capping layer and the active channel layer^13^. Choi *et al*. reported using TCAD simulation the stability is improved with flowing the current flow in ω-shape by low-resistivity capping layer^15^. In this capping structure, studies on electrical properties and stability have been progressed steadily through a simple process, but studies on various active channel layer materials and capping layer materials are still lacking.

Here, we report the change of the electrical characteristics of SZTO active channel layer among AOS candidates by varying capping layer materials from the traditional metallic materials to the oxide-based electrode materials as well as transparent conductive oxide (TCO). The electron injection or depletion phenomenon by adopting different capping layer were analyzed by using different energy band gap and work function difference. We have also implemented the NOT, NAND, and NOR logic circuits by controlling electrical characteristics simply the use of different capping layer.

## Results and Discussion

**Figure 1:**
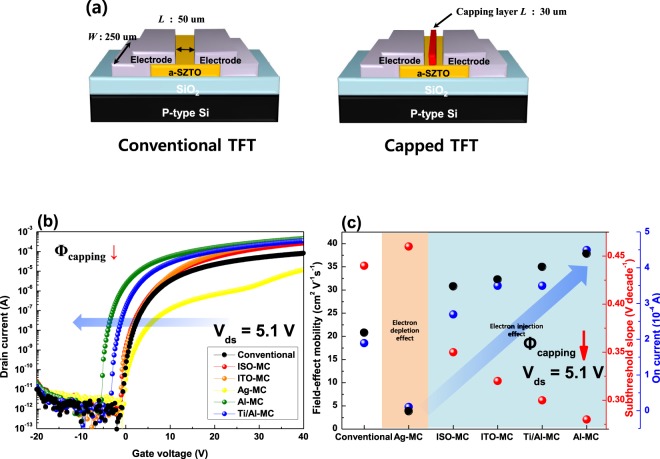
(**a**) Schematic view of conventional-TFT and capped-TFT. (**b**) Transfer characteristic and (**c**) electrical performance of TFTs with different capping layer.

Figure 1(b,c) show transfer characteristic and electrical properties of SZTO-TFT with different capping layers. Other electrical characteristics, such as threshold voltage (*V*_*th*_), on-current (*I*_*on*_), and on/off-current ratio (*I*_*on/off*_), are summarized in Table 1. The *V*_*th*_ of the TFTs were calculated, which was the gate voltage at *I*_*ds*_ of 1 nA current level. Also, the field-effect mobility (*µ*_*fe*_) was calculated by using the following Equation^9^:

1 $${\mu }_{fe}=\frac{{{\rm{Lg}}}_{{\rm{m}}}}{{\rm{W}}\,{{\rm{V}}}_{{\rm{ds}}}{{\rm{C}}}_{{\rm{ox}}}}$$

where *g*_*m*_ is the trans-conductance, *C*_*OX*_ is the oxide capacitance of the gate dielectric, *V*_*ds*_ is the drain to source voltage and *W* and *L* are channel width and length. In this manuscript we use *V*_*ds*_, *W* and *L* to 5.1 V, 250 μm and 50 μm, respectively. In the case of the capping layer, the same channel *L* value was set to extract the *µ*_*fe*_ same as in our previous study^15^. In ref. ^15^, we analyzed the current path of the capping layer and further experiment has been conducted for the channel *L*. However, the electrical characteristics were not improved when a short *L*-TFT, such as the channel length except the portion occupied by the capping layer above the channel layer, was fabricated. In addition, the current path was formed in the ω-shape, which is not due to a phenomenon, such as a short-channel effect. Therefore, we extracted *µ*_*fe*_ of the capped-TFT by fixing an *L* value of 50 μm, for the comparison of the same conventional-TFT. The capped-TFTs have the same source/drain electrode and exhibit a change in electrical properties as the only capping layer material on top of the SZTO layer changes. We have confirmed that the change of the electrical performance could be explained by two different types depending on the capping layer based on the conventional device. First, when Ag is used as the capping layer, *μ*_*fe*_, on-current (*I*_*on*_), and subthreshold slope (*SS*) are deteriorated more than those of the conventional-TFT. The deterioration of these properties is mainly due to the electron-depletion phenomenon in which electrons in the SZTO layer move to the capping layer^13^. Because of this phenomenon, degradation of electrical properties, such as *μ*_*fe*_ of 3.85 cm^2^V^−1^s^−1^, *I*_*on*_ of 1.2 × 10^−5^ A, and *SS* of 0.46 V decade^−1^ has been observed. On the contrary, the electrical characteristics tend to be improved systematically depending on the capping layer, such as ISO, ITO, Ti/Al, and Al. This effect is mainly due to the electron-injection phenomena where electrons move from the capping layer materials to the conduction band of the SZTO layer. It has been clearly observed that the electrical characteristics of the TFTs have been systematically improved of *μ*_*fe*_ (20.79 to 37.84 cm^2^V^−1^s^−1^), *I*_*on*_ (1.9 × 10^−4^ to 4.5 × 10^−4^ A), and *SS* (0.44 to 0.28 V decade^−1^) by adopting capping layer as the electron-injection phenomenon occurs. In the case of *V*_*th*_, it was confirmed that threshold voltages move from 0.65 to −4.69 V in the negative direction because of the electron injection into the SZTO layer. As a result, it was confirmed that different capping layers strongly change the electrical characteristics of the TFT, which can be analyzed by the work function difference between the channel layer and the capping layer material. We have conducted the energy band diagram analysis depending on various conductive capping layer to investigate the effect of contact potential appearing in the SZTO layer and capping layer resulting in the change of electrical performance seriously in TFTs.

**Table 1:** The electrical properties and work function of a-SZTO TFTs for different capping layer materials.

Capping layer	*V*_th_ (V)	*I*_on_ (A)	*I*_on/off_	*µ*_FE_ (cm^2^V^−1^s^−1^)	*SS* (Vdecade^−1^)	*Φ* (eV)
Conventional	0.65	1.9 × 10^−4^	1.6 × 10^9^	20.79	0.44	4.53
Ag capped	1.82	1.2 × 10^−5^	2.0 × 10^7^	3.85	0.46	4.64
ISO capped	−0.02	2.7 × 10^−4^	6.7 × 10^8^	30.79	0.35	4.49
ITO capped	−0.18	3.5 × 10^−4^	2.3 × 10^9^	32.27	0.32	4.51
Ti/Al capped	−2.35	3.5 × 10^−4^	2.9 × 10^9^	35.00	0.30	3.92
Al capped	−4.69	4.5 × 10^−4^	3.0 × 10^9^	37.84	0.28	3.79

**Figure 2:**
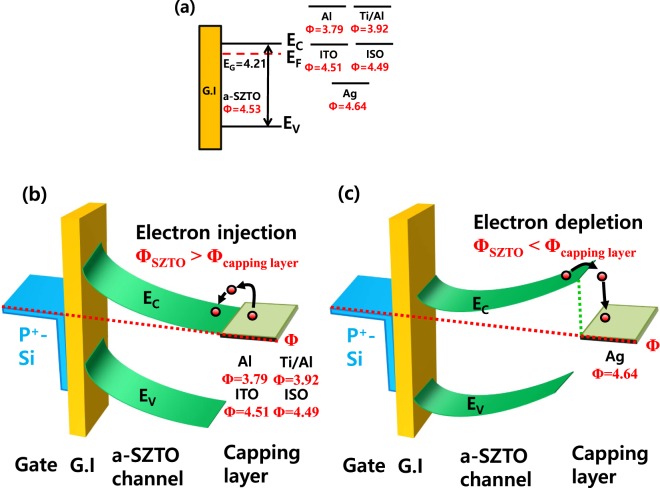
Energy band diagram of SZTO-TFTs with different capping layer in thermal equilibrium. (**a**) non-contact, (**b**) electron-injection model (when *Φ*_*SZTO*_ > *Φ*_*capping layer*_), and (**c**) electron-depletion model (when *Φ*_*SZTO*_ < *Φ*_*capping layer*_).

Figure 2 shows the energy band diagram for the SZTO-TFT depending on various capping layers with different energy band gap in thermal equilibrium state. In our previous research, we analyzed different Si content can change the energy band of ZTO system systematically by using ultraviolet photoelectron spectroscopy (UPS), high resolution electron energy loss spectroscopy (HR-EELS), and Kelvin probe force microscopy (KPFM) methods^16^. In ref. ^16^, for the ZTO system with a Si content of 0.5 wt.%, the bandgap was 4.21 eV and the work function (*Φ*) was 4.53 eV. We also measured the *Φ* of the capping layer materials using the KPFM method. The contact potential difference during the measurement was calibrated based on Pt/Ir (Tip *Φ* = 4.91 eV)^17,18^. According to KPFM results, the materials used in the capping layer showed *Φ* values of Al of 3.79, Ti/Al of 3.92, ISO of 4.49, ITO of 4.51, and Ag of 4.64 eV. In addition, the standard deviation of all capping materials was about 0.012 eV. We have extracted the energy band diagram for the two types of the electron-injection and electron-depletion as shown in Fig. 2 (b,c), respectively. When ITO, ISO, Ti/Al, and Al materials are used as the capping layer, electron-injection occurs because the *Φ* of the capping layer material is smaller than the *Φ* of the channel layer (*Φ*_*SZTO*_ > *Φ*_*capping layer*_)^13^. Therefore, it could be expected that as the *Φ* of the capping layer material becomes smaller, the movement of electrons from the capping layer to the SZTO layer becomes smooth and the electrical characteristics are improved. In the case of metal-based capping layers among the materials exhibiting electron-injection phenomena, the resistivity does not show a large difference (≈10^−6^ Ω∙cm). Nevertheless, it appears that the improvement of the electrical performance is due to the electron-injection phenomenon. However, in the case of oxide-based capping layers such as ITO and ISO, the ISO capped-TFT electron-injection phenomenon occurs easily than ITO Because the work function of ISO is smaller than that of ITO, but the resistivity is higher than that of ITO. Therefore, it should be considered that ITO capped-TFT shows better electrical performance than ISO capped-TFT due to the difference of the resistivity even though small work function. For Ag capped-TFT case, however, when the *Φ* of the capping layer is larger than the *Φ* of the SZTO layer (*Φ*_*SZTO*_ < *Φ*_*capping layer*_), electrons of the capping layer become difficult to inject into the SZTO layer because the barrier exists and rather the electrons of the SZTO layer move to the capping layer, so that the SZTO layer loses electrons and provides a lower electrical performance than the conventional-TFT. By adopting two types of electron-injection and electron-depletion capped TFTs, we have implemented two types of NOT logic circuit (or inverters).

**Figure 3:**
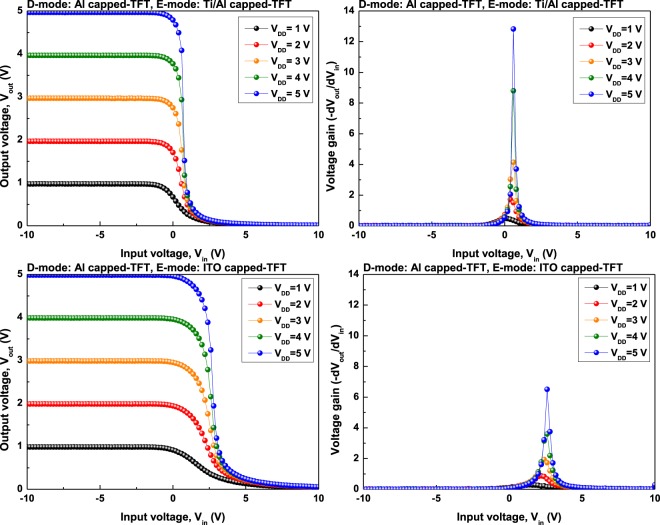
The voltage transfer characteristic (VTC) curves and voltage gain of the NOT logic circuit using different E-mode TFT, obtained for various supply voltage (*V*_*DD*_) from 1 to 5 V.

Figure 3 shows the voltage transfer characteristic (VTC) depending on the *V*_*DD*_ from 1 to 5 V of the fabricated NOT logic circuits. We fabricated the model of NOT logic circuit as a depletion-load type, and the capped-TFT with Al capping layer as the lower *V*_*th*_ value than other capped-TFT was commonly used as a depletion mode (D-mode), and the enhancement mode (E-mode) has been designed by using the capped-TFT using an ITO capping layer or Ti/Al capping layer. In general, the operation of depletion-load type NOT logic circuit can be roughly divided into three regions. In region I, when input voltage (*V*_*IN*_) is lower than the driving voltage of the E-mode TFT, *V*_*DD*_ is output to output voltage (*V*_*OUT*_). In region II, when *V*_*IN*_ is greater than or equal to the driving voltage of the E-mode TFT, the circuit has full-swing characteristics and the *V*_*OUT*_ drops to near 0 V. In the subsequent region III, the E-mode TFT exhibits an operating characteristic in which the *V*_*OUT*_ is maintained at 0 V as the TFT is fully opened^19^. The D-mode TFT always maintains the on-state when the circuit is driven as follows^20^. This is because the gate electrode and the source electrode connected each other in the case of the D-mode TFT. It can be seen that both of the fabricated NOT logic circuits exhibit full-swing characteristics. In addition, voltage gain (|δ*V*_*OUT*_/δ*V*_*IN*_|) is extracted through VTC and summarized in Table 2. It was confirmed that the voltage gain of the NOT logic circuit using the Ti/Al capped-TFT as the E-mode TFT shows a value higher than that of the ITO capped-TFT with respect to the *V*_*DD*_ change. The maximum voltage gain can be seen when Ti/Al capped-TFT and ITO capped-TFT are 12.83 and 6.51, respectively, when *V*_*DD*_ is 5 V. The difference in voltage gain strongly depends on the *SS* value of the E-mode TFT. It was observed that the depletion-load type NOT logic circuit is sensitive to the *SS* value rather than the *μ*_*fe*_ of the E-mode TFT^21^. Therefore, we can analyze that Ti/Al capped-TFT with better *SS* value can show higher voltage gain value in this case. We have fabricated more complex NAND and NOR logic circuits using Ti/Al capped-TFT, which exhibits high performances in NOT logic circuit.

**Table 2:** The maximum voltage gain values of the NOT logic circuit for *V*_DD_ from 1 to 5V.

*V*_*DD*_ E-mode TFT	1 V	2 V	3 V	4 V	5 V
Ti/Al capped-TFT	0.48	1.71	4.14	8.79	12.83
ITO capped-TFT	0.28	0.87	1.93	3.60	6.51

**Figure 4:**
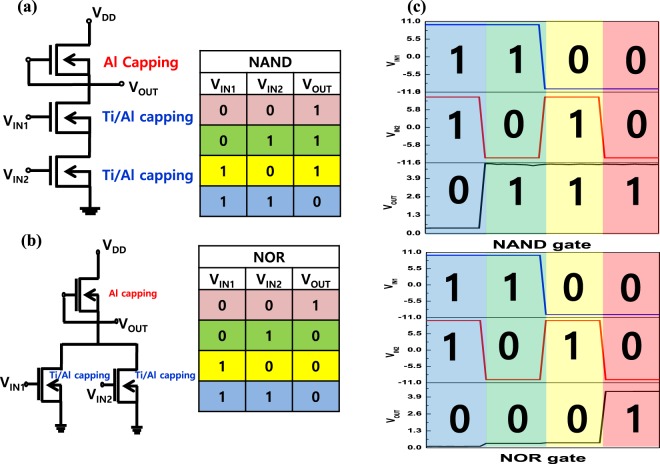
NAND (**a**) and (**b**) NOR (**b**) logic circuits, showing circuit diagram and logic table. (**c**) *V*_*OUT*_ cycling of NAND and NOR circuits, for prescribed *V*_*IN*_ sequence.

Figure 4 shows the circuit diagram, truth table, and output characteristics of NAND and NOR fabricated. We connected the previously configured NOT logic simply by wire bonding using an additional Ti/Al capped-TFT as the E-mode-TFT2. In the case of the NAND logic circuit, the two E-mode-TFTs are connected in series and the NOR logic circuit can be connected in parallel^22^. In the case of wire bonding, we may report on simple output characteristics in this manuscript, although it may affect the logic circuit characteristics by connecting each device using additional wiring. However, these problems will not be serious because they can be simply processed by forming independent gates. In addition, after *V*_*DD*_ was fixed at 5 V, we applied a voltage of −10 V and 10 V, which could show sufficient operating characteristics for each of *V*_*IN1*_ and *V*_*IN2*_. The *V*_*OUT*_ was output close to the *V*_*DD*_ setting value of 5 V (digital signal of “1 (high)”), and it was observed that the *V*_*OUT*_ drop to 0 V (digital signal of “0 (low)”) when the E-mode TFTs were operated. As a result, when we configure the NAND and NOR logic circuits simply using different capping layer, it was confirmed that the same operation as the truth table is normally observed.

## Conclusion

In summary, we have investigated the changes in electrical properties for TFTs with different capping layer materials. It has been observed that the change of electrical characteristics is sensitively due to the difference in work function of the capping layer as compared with the conventional-TFT. Based on the work function of the channel layer, electron-injection and electron-depletion have been found, depending on the work function of the capping layer materials. As the work function of the capping layer becomes smaller, the electrical performance of TFT has been improved easily by electron-injection phenomena. In addition, when the best electrical characteristics were shown, TFTs using Al as a capping layer showed *μ*_*fe*_ of 37.84 cm^2^V^−1^s^−1^, *I*_*on*_ of 4.5 × 10^−4^ A, and *SS* of 0.28 Vdecade^−1^. Since the electrical characteristics could be changed sensitively depending on the change of the capping layer, we could easily fabricate the logic circuit. NOT logic circuit showed high voltage gain of 12.83 at *V*_*DD*_ = 5 V when using Ti/Al capped-TFT as E-mode. In the case of NAND and NOR logic circuits, it is confirmed that the output characteristics according to the inputs are driven in the same manner as the truth table. These results can be directly applied to next-generation thin film logic circuits and electronic devices.

## Methods

### Device fabrication

All TFTs choose the substrate that heavily doped p^+^-type silicon wafer (resistivity 0.001–0.002 Ω/cm), which has 200 nm thick SiO_2_depositedby thermal oxidation process. The substrate was cleaned in acetone, methanol, and deionized water using conventional ultrasonic cleaning method. The powder mixture of 99.99% pure ZnO, SnO_2_, and SiO_2_ was used as a SZTO (SiO_2_: ZnO: SnO_2_ = 0.5: 65: 35 wt.%) target. We fixed the thickness of the channel layer to 24 nm to analyze the effect of the capping layer. The SZTO channel layer was deposited using RF-sputtering process. The deposition condition was RF power density of 60 W, working pressure of 3 mTorr, and the oxygen and argon ratio of 40: 1 at room temperature. The SZTO-TFTs with inverted staggered bottom gate and top source/drain electrode structure were fabricated by conventional photo-lithography and wet-etching. After channel patterned, SZTO thin films were annealed at 500 ^o^C in air ambient for 2 hrs. Thereafter, the source/drain electrodes were commonly fabricated using the Ti/Al material by lift-off process and e-beam/thermal evaporation. The capping layer was fabricated by changing the material using the same method as the source/drain process. The materials used in the capping layer are Ag, ISO, ITO, Ti/Al, and Al, respectively. The conventional TFT *W/L* ratio of the channel was 250 μm/50 μm and capping layer *L* was 30 μm, as shown in Fig. 1(a).

### Characterization

The work function of each capping layer materials were measured by using Kelvin probe force microscopy (KPFM) The electrical characteristics were measured using a semiconductor parameter analyzer (EL 423, ELECS Co.) at room temperature in a dark box.NAND and NOR logic circuit performance was characterized by using a triple output DC power supply (E3631A, Keysight Co.) to supply the input voltage *V*_IN_, with *V*_OUT_ recorded by the semiconductor parameter analyser.
